# High-throughput rapid amplicon sequencing for multilocus sequence typing of *Mycoplasma ovipneumoniae* from archived clinical DNA samples

**DOI:** 10.3389/fvets.2024.1443855

**Published:** 2024-07-31

**Authors:** Isaac Framst, Rebecca M. Wolking, Justin Schonfeld, Nicole Ricker, Janet Beeler-Marfisi, Gabhan Chalmers, Pauline L. Kamath, Grazieli Maboni

**Affiliations:** ^1^Department of Pathobiology, Ontario Veterinary College, University of Guelph, Guelph, ON, Canada; ^2^Washington Animal Disease Diagnostic Lab, Washington State University, Pullman, WA, United States; ^3^Public Health Agency of Canada, Guelph, ON, Canada; ^4^School of Food and Agriculture, University of Maine, Orono, ME, United States; ^5^Athens Veterinary Diagnostic Laboratory, College of Veterinary Medicine, University of Georgia, Athens, GA, United States; ^6^Department of Infectious Diseases, College of Veterinary Medicine, University of Georgia, Athens, GA, United States

**Keywords:** long-read sequencing, short-read sequencing, bacterial typing techniques/methods, sheep respiratory disease, *Mycoplasma ovineumoniae*

## Abstract

**Introduction:**

Spillover events of *Mycoplasma ovipneumoniae* have devastating effects on the wild sheep populations. Multilocus sequence typing (MLST) is used to monitor spillover events and the spread of *M. ovipneumoniae* between the sheep populations. Most studies involving the typing of *M. ovipneumoniae* have used Sanger sequencing. However, this technology is time-consuming, expensive, and is not well suited to efficient batch sample processing.

**Methods:**

Our study aimed to develop and validate an MLST workflow for typing of *M. ovipneumoniae* using Nanopore Rapid Barcoding sequencing and multiplex polymerase chain reaction (PCR). We compare the workflow with Nanopore Native Barcoding library preparation and Illumina MiSeq amplicon protocols to determine the most accurate and cost-effective method for sequencing multiplex amplicons. A multiplex PCR was optimized for four housekeeping genes of *M. ovipneumoniae* using archived DNA samples (*N* = 68) from nasal swabs.

**Results:**

Sequences recovered from Nanopore Rapid Barcoding correctly identified all MLST types with the shortest total workflow time and lowest cost per sample when compared with Nanopore Native Barcoding and Illumina MiSeq methods.

**Discussion:**

Our proposed workflow is a convenient and effective method for strain typing of *M. ovipneumoniae* and can be applied to other bacterial MLST schemes. The workflow is suitable for diagnostic settings, where reduced hands-on time, cost, and multiplexing capabilities are important.

## 1 Introduction

Bronchopneumonia is a population-limiting disease of bighorn sheep (BHS), *Ovis canadensis*, across western North America. *Mycoplasma ovipneumoniae*, the primary etiologic agent of this disease, is transmitted to BHS through contact with a herd of domestic sheep and goats, which are carriers of the pathogen (1, 2). *M. ovipneumoniae* demonstrates a high degree of genetic diversity across its host range (1, 3). The genetic diversity of *M. ovipneumoniae* is high in domestic sheep, indicating their role as a significant reservoir and source of infection, while in BHS, it is low, suggesting spillover as the primary transmission source (1). Indeed, state reconstruction of ancestral sequences from multi-locus sequence typing (MLST) sequences confirmed domestic sheep as the primary source of infection for BHS, emphasizing the importance of strain typing to map transmission dynamics (4). In BHS, an initial outbreak of fatal bronchopneumonia is often followed by recurring fatal outbreaks in lambs. Recurrent outbreaks have been observed from 2 to 15 years after the initial spillover (2, 5–7). Recent evidence suggests that there may be no cross-strain immunity, leaving surviving animals susceptible to infection (4, 8). To reduce the likelihood of spillover events, federal and state agencies have implemented policies focused on the spatial separation of domestic and wild sheep (9). Increased sampling efforts in the western US and Canada have recently been undertaken to find the wider prevalence of *M. ovipneumoniae* in 10 states and three provinces (10).

DNA-based strain typing is used to document the invasion, persistence, and transmission of *M. ovipneumoniae* in these populations (7). A previously developed MLST scheme targeting four gene fragments, namely, the 16-23S intergenic spacer region (IGS), 16S rRNA region (LM), RNA polymerase β-subunit gene (*rpoB*), and DNA gyrase subunit-β gene (*gyrB*), has demonstrated strong differential typing capability in over 600 samples and 270 strain types (1, 4). Creating a conventional database of alternative alleles is impractical due to the rapid emergence of new strains and the extensive diversity of novel types (1, 8, 10). In the current Sanger workflow, the four gene fragments are concatenated and then compared pairwise with previously stored type sequences. The definition of a strain is established based on its similarity to stored types using a specific threshold of four base pairs (1). In cases where *rpoB* and *gyrB* do not amplify or are unavailable, strains are denoted by their IGS length, which is consistent with historical typing methods in use prior to the current MLST scheme (4, 7).

The current MLST laboratory process uses a nested singleplex polymerase chain reaction (PCR), and Sanger sequencing applies to each locus. This method is laborious and expensive if processing a large number of samples (11). Oxford Nanopore Technologies (ONT) sequencing has recently been used for singleplex and multiplex MLST (12–16). This method uses a small, low-cost sequencing device, resulting in real-time multiplexing and high-throughput sequencing. Amplicon sequencing using ONT has been previously validated for antimicrobial resistance genotyping of *Neisseria gonorrhoeae* (14). This and other similar workflows were estimated to cost approximately 100 times less than Sanger sequencing for large sample sets (14, 17).

Native Barcoding library preparation of ONT is recommended for amplicon sequencing due to its higher read accuracy and preservation of the full-length amplicon (18). Alternatively, Rapid Barcoding library preparation is faster and less expensive; however, high-throughput and raw read accuracy are reported to be reduced (19). An amplicon-specific protocol for Rapid Barcoding is not provided by ONT, although several studies have used that kit for sequencing multiplexed amplicons with a high degree of accuracy (12–15). Based on these successes, Rapid Barcoding library preparation is expected to be well-suited for diagnostic settings because of the short library preparation time, flexible multiplexing options, accuracy, and low cost.

We aimed to develop a next-generation sequencing workflow by multiplex PCR followed by Rapid Barcoding Nanopore sequencing using archived DNA from clinical samples. We also compared the speed and accuracy of the optimized Rapid Barcoding workflow with other Nanopore library preparation methods and Sanger and Illumina sequencing.

## 2 Materials and methods

### 2.1 Samples

Archived DNA samples (*n* = 88) were provided by the Washington Animal Disease Diagnostic Laboratory (WADDL) at Washington State University (Pullman WA, United States) (Supplementary 1). The DNA samples originating from bighorn sheep field samples were submitted to WADDL between 2011 and 2016 for diagnostic testing as part of a previously published study (1, 4). The presence of *M. ovipneumoniae* DNA was determined by qPCR at WADDL (20). DNA samples were stored at −20°C in a non-defrosting freezer until processing. Storage time was between 3 and 12 years. Archived DNA samples with less than 10 μL of total volume were discarded from the study. *M. ovipneumoniae* strain Y98 was purchased from the American Type Culture Collection (ATCC 2941 – Y98, domestic sheep, 1976, NCBI BioProject PRJNA253514) for use as a control sample. Bacterial culture and DNA extraction of the reference strain were performed as previously described (21). Sequence typing of samples was previously determined using nested singleplex PCR assay and Sanger sequencing by WADDL (4). Since current *M. ovipneumoniae* strain typing workflows use Sanger sequencing, new methods were compared with the results obtained by Sanger sequencing. For initial PCR amplification and NGS sequencing, 68 samples of sufficient volume were used. A subset of 24 samples was selected for additional sequencing runs. This subset was chosen to reflect the diversity of strain types and cycle thresholds of the larger set.

### 2.2 PCR assays

#### 2.2.1 Singleplex PCR

Nested singleplex PCR was performed using primers targeting LM, IGS, *rpoB,* and *gyrB* loci (Supplementary 2A) (4). Cycling conditions were modified for the Phusion Flash HiFi all-in-one master mix (Thermofisher, Waltham, MA, United States) (Supplementary 2B). External nested PCR reactions were performed for IGS, *rpoB,* and *gyrB* targets, and then, 1 μL was transferred to the inner nested reaction (Supplementary 2C). A single PCR reaction was used for LM. The nuclease-free water sample was used as a negative control in each PCR run. Detailed singleplex PCR cycling conditions are presented in Supplementary 2.

#### 2.2.2 Multiplex PCR

The internal and external primers were pooled in equimolar concentrations of 0.2 μM for a 50 μL PCR reaction (Supplementary 3A) (4). A three-step PCR protocol was then optimized using a series of 2-fold serial dilutions of Y98 pure isolate DNA (ATCC 2941 – Y98 strain) and a subset of five samples. Optimal annealing temperature and primer concentration were determined experimentally (Supplementary 3B). Singleplex and multiplex PCR products were stored at −20°C until sequencing library preparation.

#### 2.2.3 Gel electrophoresis

For singleplex and multiplex PCR products, 1.5 and 2% (w/v) of agarose gels were used, respectively. Gels were prepared in-house using a 1X lithium acetate borate buffer solution (Sigma–Aldrich, Burlington, MA, United States), SYBR safe DNA gel stain (Invitrogen, Waltham, MA, United States), and a 100–1,000 bp DNA marker (Invitrogen, Waltham, MA, United States), and all samples were loaded using TriTrack DNA loading dye (ThermoFisher Scientific, Waltham, MA, United States). Gels were run in the buffer solution at 120 V and then imaged using a GelDoc Go (Bio-Rad, Hercules CA, United States).

### 2.3 Illumina sequencing

A total of 24 samples of four nested singleplex PCR products were submitted to the Advanced Analysis Centre (AAC) Genomics facility (University of Guelph, Guelph, ON, CA) for sequencing on an Illumina MiSeq platform (Illumina Technologies, San Diago, CA, United States). The facility used a modified 16S ribosomal RNA gene amplicon protocol (Illumina Part # 15044223 Rev. A), with custom primers and a maximum insert size of 550 bp (Supplementary 5). Sequences were returned as demultiplexed FASTQ files.

### 2.4 ONT sequencing

Three ONT sequencing experiments were conducted to determine the optimal library preparation method and flow cell configuration (Table 1). Before library preparation, PCR products were quantified by a Qubit Fluorometer using the dsDNA broad range kit (Invitrogen, Waltham, MA, United States) and diluted without purification according to the ONT library preparation protocol.

**Table 1:** Description of Oxford Nanopore sequencing experiment conditions.

Experiment No.	Flow cell chemistry^a^	Library preparation^b^	Flow cell^c^	Library sample size^d^	Runtime (h)
Experiment 1	R9.4.1	Rapid	New	68	16
			Washed	24	16
Experiment 2 Experiment 3	R10.4.1	Native	New	24	16
			Washed	24	16

All sequencing runs were conducted using a minION Mk1B device: Oxford Nanopore Technologies (ONT).

a Specific flowcell chemistry used.

b Library preparation method. Rapid: Nanopore Rapid Barcoding (SQK-RBK110/114.96); Native: Nanopore Native Barcoding (SQK-NBD114.96).

c New: Unused flowcell from ONT, washed: washed flow cell reused from previous sequencing run using EXP-WSH004 wash kit.

d Number of samples barcoded in the prepared library. The same 24 samples were included in all libraries.

#### 2.4.1 ONT Experiment One

ONT Experiment One determined the suitability of ONT Rapid Barcoding library preparation for multiplexed amplicons using ONT R9.4.1 flow cell, Rapid Barcoding library preparation kit, and flow cell wash kit (EXP-WSH004) (Oxford Nanopore Technologies, Oxford, UK). PCR products were quantified by a Qubit Fluorometer using the dsDNA broad range kit (Invitrogen, Waltham, MA, United States) and diluted without purification according to the ONT library preparation protocol (RBK_9126_v110_revO). Then, 68 multiplex PCR products were barcoded according to the SQK-RBK110.96 protocol. An R9.4.1 flow cell was loaded with 400 ng of DNA library. Following a 16-h runtime, the flow cell was washed using the EXP-WSH004 kit and immediately loaded with a second library containing a subset of 24 barcoded samples from the preceding run. The second library (washed flow cell) was sequenced for 16 h.

#### 2.4.2 ONT Experiment Two

ONT Experiment Two evaluated the Native Barcoding library preparation kit, with the most current ONT R10.4.1 flow cell as recommended by ONT compared with ONT Experiment One. A total of 24 multiplex PCR products were barcoded according to the SQK-NBD114.96 protocol (ONT protocol NBA_9170_V114_REVG_15SEP2022, 2023). Following a 16-h runtime, the flow cell was washed using the EXP-WSH004 kit and immediately loaded with a second library of the same 24 samples. Libraries were differentially barcoded to avoid barcode contamination between the runs. The second library was prepared using the same method as the first run and sequenced for 16 h.

#### 2.4.3 ONT Experiment Three

ONT Experiment Three was performed identically to ONT Experiment Two by another technician to control human error.

#### 2.4.4 ONT sequencing run parameters

All sequencing runs used minION Mk1B instrument and minKNOW v23.7.15 (ONT) operating software. The minimum read length was set to 20 bp; real-time basecalling was turned off since Dorado real-time base calling was unsupported when the experiment was conducted. MinKNOW read output was set to “.POD5,” to collect raw signal data, active channel selection was turned on, and “reserve pores” were turned off to maximize initial high throughput. Runtime was set to 16 h in all experiments. For new and washed flow cells, a flow cell check was conducted immediately prior to loading the library using the “flow cell check” option on the minKNOW software homepage. Flow cells with fewer than 800 new or 400 washed active pores were not used.

### 2.5 Data analysis

#### 2.5.1 ONT analysis

**Figure 1:**
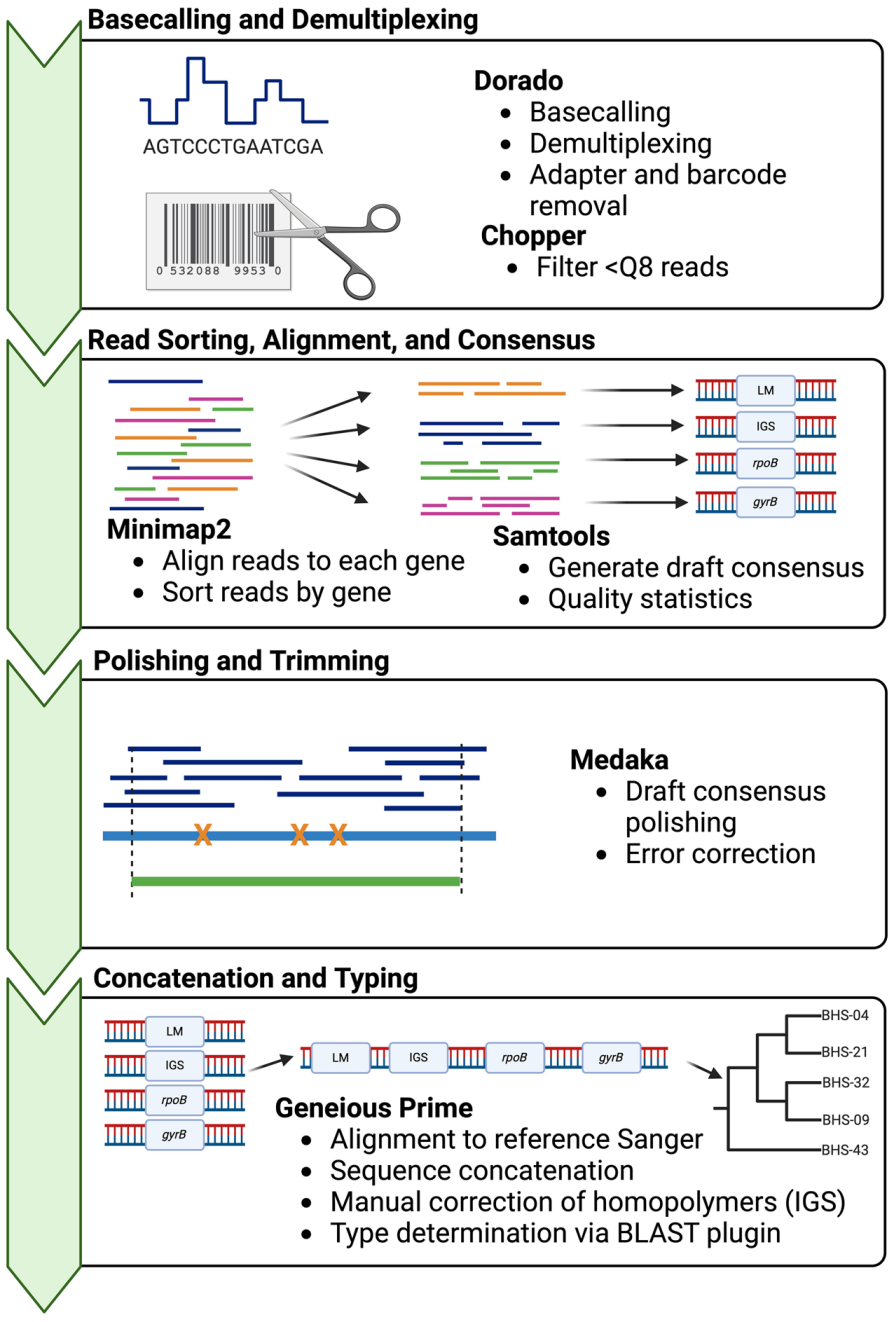
Bioinformatics workflow for obtaining *Mycoplasma ovipneumoniae* MLST sequences from multiplexed Oxford Nanopore reads. Squiggle data (fast5/pod5) are basecalled in real-time or post-run using Dorado basecaller. The resultant sequence reads then undergo two deconvolution steps: separation of reads according to barcode using Dorado demux, followed by alignment of reads to the target reference sequences with minimap2. Multiple loci are present in one barcoded sample (multiplex PCR product) and must be binned by the target. The consensus sequences are called from each alignment using SAMtools and then concatenated for typing in Geneious Prime. Phylogenies are built using concatenated sequences in RAxML (22). Strain types are determined by the pairwise identity of the concatenated sequence to other archived types.

Basecalling was performed with Dorado v0.3.0^1^ using the “fast” model with demultiplexing. Then, the demultiplexed FASTQ files were submitted to a custom analysis pipeline (Figure 1). In brief, basecalled reads were demultiplexed and trimmed using GUPPY v.6.4.8 (ONT), reads below a quality score of 8 were removed using Chopper v0.5.0 (23), and then reads were aligned to the four reference genes, i.e., a deconvolution step, using Minimap2 v2.24 (24). The resultant alignments were sorted and indexed, and then, alignment statistics were generated, including depth and number of reads mapped using SAMtools v.1.17 (25). Consensus sequences were “called” using SAMtools consensus with default calling (Bayesian mode with quality-aware mapping). Draft consensus sequences were polished using Medaka v1.8.1 (ONT), to produce final output sequences. If the sequencing depth of one or more amplicons in a sample was <50x, the sample was excluded from downstream analyses. Homopolymer errors in IGS were manually corrected after polishing by adding a T at position 113, to correct the sequence to 8 Ts. A shell script for this pipeline is provided in Supplementary 4. Sequences were imported into Geneious Prime for final typing (Supplementary 6).

#### 2.5.2 Illumina analysis

Forward and reverse reads were imported into Geneious Prime v2023.2.1 (Dotmatics). The average read quality and number of reads were recorded using the Geneious statistics panel view for each read group. Reads were paired by name and trimmed using BBDuk v1.0 (BBMap – Bushnell B.^2^) via the Geneious plugin and then aligned to the reference sequence within Geneious. Consensus sequences were generated from each alignment and compared with the corresponding reference Sanger sequence using the Geneious local alignment tool. The pairwise identity and number of mismatches were recorded. Finally, LM, IGS, and *gyrB* were concatenated for typing (Supplementary 6).

#### 2.5.3 Quality and accuracy determination of consensus sequences

Consensus sequences, which are representative sequences of each amplicon, were generated by Bayesian estimation of the true base at each alignment position using the SAMtools consensus module with quality-aware mapping. The accuracy and quality of the consensus sequences from each method were characterized by (i) sequencing coverage of each target locus, (ii) the number of reads aligned to each reference gene sequence, (iii) the percent identity of consensus sequence and corresponding Sanger sequence, and (iv) the number of mismatches between the consensus and the Sanger sequence. Gaps in the consensus sequence were replaced with Ns and treated as mismatches. The Q-score average read quality for Illumina and ONT runs was recorded using phred-33 encoding.

### 2.6 Statistical analyses

A linear regression model was constructed to determine the relationship between coverage and mismatches. The “lm” function in R was used with the number of mismatches as the response variable and coverage as the predictor variable (26). The *F*-test statistic with an associated *p*-value was used to determine the significance of the relationship from the model summary. Tukey’s honestly significant difference test was used to determine the level of independence of errors between genes and runs. Statistical significance was set at a *p*-value of ≤ 0.05.

## 3 Results

### 3.1 Singleplex and multiplex PCR

A total of 68 samples had >10 μL of reaction and were used for PCR and sequencing optimization. A singleplex nested PCR was used to amplify the four MLST loci for Illumina sequencing (Supplementary 2A–C). After gel electrophoresis, distinct bands of the expected sizes were visible for all targets (Supplementary 3C). The multiplex PCR assay was optimized for the amplification of the four MLST loci in a single 50 μL reaction. After electrophoresis, the optimized multiplex PCR produced four distinct bands of 490 bp, 470–510 bp, 547 bp, and 680 bp corresponding to LM, IGS, *rpoB,* and *gyrB,* respectively (Supplementary 3D). Fragments from external nested primers were not visible.

### 3.2 Illumina sequencing

Illumina sequencing generated full-length read pairs for IGS, LM, and *gyrB* within 48 h. Raw read and consensus quality for Illumina were higher than all ONT methods (Tables 2, 3). Illumina consensus sequences were obtained for LM, IGS, and *gyrB* loci; however, *rpoB* (680 bp) consensus sequences could not be resolved because there was no overlap for pairing the forward and reverse reads due to the maximum insert size of 550 bp. The resultant pairings were missing the middle 130 bp of the amplicon. Therefore, downstream analyses omit *rpoB* for Illumina data.

**Table 2:** Oxford Nanopore and Illumina sequencing run metrics.

Experiment	Flow cell chemistry^a^	Library preparation^b^	Flow cell^c^		Number of reads (M)		Read quality^g^		% Reads > Q20^h^
				Active pores^d^	Pre-filtering^e^	Post-filtering^f^	Pre-filtering^e^	Post-filtering^f^	
Experiment 1	R9.4.1	Rapid	New	1,426	5.66	2.41	12.0	16.6	38.3%
			Washed	935	2.95	1.45	12.5	16.3	38.5%
Experiment 2	R10.4.1	Native	New	1,646	6.53	4.00	13.6	17.6	38.3%
Experiment 3	R10.4.1	Native	New	1,092	7.87	5.01	12.9	15.7	30.6%
			Washed	491	3.40	1.99	12.5	16.0	31.4%
Illumina^i^	Illumina	Modified 16S^i^	N/A	N/A	8.32	8.00	30.4	34.2	81.2%

a Nanopore flow cell chemistry used.

b Type of kit used for library preparation. Rapid: Rapid Barcoding 96 (SQK-RBK110-96 for R9.4.1; SQK-RBK114.96 for R10.4.1); Native: Native Barcoding kit (SQK-NBD114.96).

c New: New flow cell from ONT, washed: washed flow cell reused from previous sequencing run using EXP-WSH004 wash kit.

d Number of active pores at the beginning of the sequencing run following flow cell loading.

e Metric calculated immediately post-basecalling before demultiplexing or read filtering.

f Metric calculated post-filtering, reads Q8.0 and below removed before calculation.

g Read quality reported as an average Q-score (phred33).

h Percent of total reads post-filtering with quality score > 20.

i Illumina sequencing performed using modified 16S rDNA library preparation with custom primers (Advanced Analysis Center, Guelph, Canada).

**Table 3:** Quality of Oxford Nanopore and Illumina consensus sequences post-alignment for samples with >50× coverage.

Experiment No.^a^	Flow cell^b^	No. of samples^c^	Average coverage^d^	No. reads mapping to reference^e^	Percent identity to Sanger^f^	Number of mismatches^g^	% types correctly identified
Experiment 1	New	60/68	2,037x ± 1998	3,956 ± 3,833	100% ± 0.001%	0.0 2 ± 0.19	100%
	Washed	21/24	4,803x ± 6,530	8,753 ± 11,343	100%	0	100%
Experiment 2	New	23/24	13,058x ± 13,470	13,525 ± 14,253	99.1% ± 1.3%	3.48 ± 5.59	62%
Experiment 3	New	24/24	38,293x ± 35,904	39,017 ± 37,751	99.0% ± 0.1%	3.64 ± 3.63	71%
	Washed	18/24	13,972x ± 15,120	14,195 ± 15,963	99.1% ± 0.001%	2.59 ± 5.24	83%
Illumina^h^	New	20/24	130,674x ± 57,640	130,674 ± 57,640	100% ± 0.001%	0.05 ± 0.37	N/A

Averages reported are aggregated for *gyrB*, IGS, LM, and *rpoB* for 24 samples, ± standard deviation.

a Experiment 1: Nanopore Rapid Barcoding library preparation, 16 h runtime. Experiments 2 and 3: Nanopore Native Barcoding library preparation, 16 h runtime.

b New: New flow cell from ONT, washed: washed flow cell reused from previous sequencing run using EXP-WSH004 wash kit.

c Number of samples over 50x coverage included in analysis/number of samples sequenced.

d Coverage of target loci, averaged for all samples over 50x coverage. Per-locus coverage information is available in Supplementary 7.

e Number of reads aligned to the corresponding Sanger sequence reference, reported as an average for all loci and samples in the run.

f Percent identity to the Sanger sequence reference of the same sample.

g Number of bases that do not match the same position in the Sanger sequence.

h Illumina sequencing conducted by a third party (Advanced Analysis Center, Guelph, Canada). N/A No data for rpoB (680 bp) were presented because of the 550 bp limit by Illumina sequencing. Therefore, strain type is indeterminate.

### 3.3 ONT sequencing

#### 3.3.1 Run quality

Run quality and raw read statistics are shown in Table 2. The average read quality for all ONT runs was similar before and after filtering. The highest run yield, 7.87 Gb, was from the R10.4.1 new flow cell (ONT Experiment Two). The number of active pores decreased by approximately 700 after a 16-h runtime and a washing step. The throughput of washed flow cells from ONT Experiments One and Three was reduced by approximately half for the same 16-h runtime; however, the read quality was similar to the first run. In ONT Experiment Two, flow cell washing was unsuccessful due to the formation of air bubbles over the sensor array, which irreversibly damaged the pores, leaving fewer than 100 active pores.

#### 3.3.2 ONT bioinformatics pipeline

A custom bioinformatics pipeline was constructed using open-source tools (Figure 1). The bioinformatics analysis of the 24 samples took 30–50 min from read filtering to final output, depending on the number of total reads (10 CPU cores, 32 GB memory). Polishing of consensus sequences using Medaka increased the agreement with Sanger sequences, especially for low-coverage samples.

### 3.4 Comparison between alignment and consensus sequence statistics

Consensus sequences were called using alignments generated for each gene and assessed for quality and accuracy (Table 3). The depth of coverage varied across Illumina and ONT runs (130,674x ± 57,640 and 16,699x ± 24,438, respectively); the highest coverage, 38,293x ± 35,904x, was observed in ONT Experiment Three (Native Barcoding with R10.4.1 new flow cell, *n* = 24) and the lowest coverage, 2,037x ± 1,998x, was observed in ONT Experiment One (Rapid Barcoding, R9.4.1 new flow cell, *n* = 68). Some samples were excluded from downstream analysis in ONT Experiment Three (washed) (*n =* 6 removed) due to <50x coverage even though the average depth for the run was high (1,397x ± 15,120x). The number of reads aligned to each gene closely correlated with coverage (Table 3), where ONT Experiment One had the smallest average number of reads per sample and ONT Experiment Three had the largest average number of reads. For ONT Experiment One (Rapid Barcoding, R9.4.1), the number of reads was approximately double the average coverage, while in ONT Experiments Two and Three (Native Barcoding, R10.4.1 flow cell), the number of reads was equal to the coverage. This highlights the technical differences in library preparation methods and the effect of read length.

Consensus sequences with coverage greater than 50x were compared with the corresponding Sanger sequences (Table 3). Consensus sequences in ONT Experiment One were identical to the corresponding Sanger sequences of the same samples with 100% identity, resulting in all strain types being identified. ONT Experiments Two and Three consistently shared 99% identity with the corresponding Sanger sequences, and there were more mismatches to Sanger sequences than ONT Experiment One. A linear regression analysis of mismatches for ONT Experiments Two and Three did not show a relationship between the coverage and the number of mismatches to the corresponding Sanger sequences (*R*^2^ = 0.0001648, *F*_1, 286_ = 0.04713, *p* = 0.8283). Mismatches in ONT Experiments Two and Three occurred more often in *gyrB* and *rpoB* targets, and samples with mismatches in one locus were more likely to have mismatches in other loci (Tukey’s HSD, *p* < 0.05) with a bimodal distribution of mismatches. The number of mismatches was similar between ONT Experiments Two and Three, and *rpoB* consistently had the most mismatches. The mismatches predominantly clustered at positions that corresponded to regions, where nucleotide substitutions indicative of other strain types were observed. Only 72% (*n* = 47 of 65) of samples were correctly typed for ONT Experiments Two and Three (Table 3).

#### 3.4.1 Turnaround time

**Figure 2:**
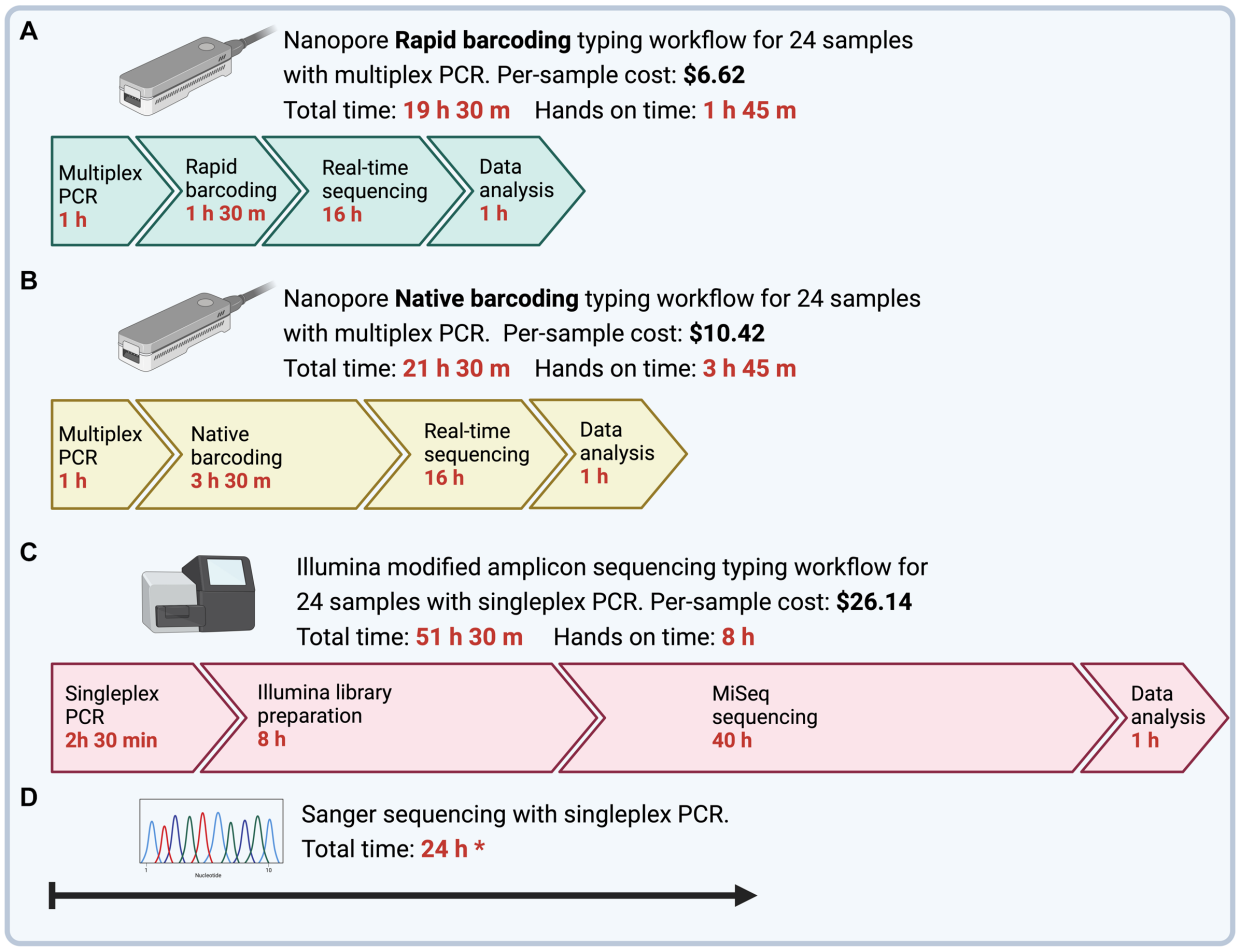
Time and cost to complete *Mycoplasma ovipneumoniae* MLST workflows from PCR to final typing of sequences. To emphasize differences in hands-on time between methods, timeline steps are not drawn to scale. **(A)** Four genes are amplified using a single multiplex PCR and barcoded using Nanopore Rapid Barcoding library preparation. The subsequent library is sequenced using the minION device with a new or washed flow cell. **(B)** Four genes are amplified using a single multiplex PCR and barcoded using the Nanopore Native Barcoding library preparation. The subsequent library is sequenced using the minION device with a new or washed flow cell. All nanopore reads are trimmed and filtered to remove adapters and low-quality regions, and then reads are sorted by MLST loci. The resultant alignment is used to call a draft consensus and then polished to correct potential errors. **(C)** Four genes are amplified separately using a nested singleplex PCR assay with seven total reactions and prepared for sequencing using the Illumina 16S metagenomic sequencing library preparation with primers modified for the *M. ovipneumoniae* MLST scheme. A third-party laboratory sequenced the subsequent library with an Illumina MiSeq, 600-cycle flow cell. Illumina reads are trimmed and filtered and then aligned to the respective reference gene for consensus calling. **(D)** Four genes are amplified separately using a nested singleplex PCR assay with seven total reactions and sent for Sanger Sequencing by an offsite facility. All prices are per sample, including the cost of library preparation and flow cell, assuming multiplexed runs BioRender.

The time to obtain MLST sequences from DNA samples was determined as the time from PCR preparation to final typing output (Figure 2). Multiplex PCR with ONT Rapid Barcoding library preparation (ONT Experiment One) had the shortest turnaround time of 19.5 h, of which 1 h 45 min was hands-on time. The Illumina sequencing workflow with singleplex PCR could be completed within 52 h with 8 h of hands-on time. However, the turnaround time for Illumina sequencing depended on third-party services, which took 4 to 8 weeks for us to receive the sequencing results.

#### 3.4.2 Comparison of ONT with Illumina

ONT sequencing generated fewer reads and coverage than Illumina sequencing (Table 3). Sequences obtained from ONT Experiment One (Rapid Barcoding, R9.4.1 flow cell) and Illumina had 100% identity with Sanger sequences. ONT Experiments Two and Three (Native Barcoding, R10.4.1 flow cell) showed lower percent identity with Sanger sequences than the other experiments (Table 3). ONT Experiment One (Rapid Barcoding, R9.4.1 flow cell new and washed) was the only method that correctly identified all strain types. Illumina consensus sequences were identical to Sanger sequences for 23 of the 24 samples for LM, *gyrB, and IGS,* with three mismatches occurring in a single sequence (IGS, WADDL #00126). Illumina sequencing failed to generate *rpoB* sequences. Since *rpoB* could not be recovered fully with the Illumina method, strain types could not be determined.

## 4 Discussion

In this study, we compared the efficiency of different sequencing approaches for strain typing of *M. ovipneumoniae*, including ONT, Illumina, and Sanger sequencing. We optimized and validated a workflow using multiplex PCR and Rapid ONT sequencing for strain typing of *M. ovipneumoniae* from DNA samples. Furthermore, we developed a custom bioinformatics pipeline to deconvolute and align reads, generate a consensus, and error-correct the final consensus sequences.

Illumina sequences had the highest quality read and consensus Q-scores; however, due to the maximum insert size of 550 bp, the full-length *rpoB*, at 680 bp, could neither be paired nor aligned. To maintain backward compatibility with the Sanger scheme, we followed the Illumina method, which was insufficient for all loci, and it was more expensive and time-consuming. However, in a similar study, multiplex PCR of four genes for MLST of *M. genitalium* decreased the cost of Illumina library preparation, and all target fragments were under 500 bp in length (27). This approach could be useful for *M. ovipneumoniae* MLST in diagnostic laboratories that already use Illumina but would require redesigning of the *rpoB* primer set to reduce amplicon length, which risks the removal of relevant bases.

The Rapid and Native Barcoding library preparations from ONT were compared to determine their suitability for multiplex amplicon sequencing. We removed samples that had less than 50x sequencing depth at one or more loci from downstream analysis. The Rapid Barcoding library approach identified 100% of strain types despite having a lower total yield and lower per-loci depth than Native Barcoding (72% identified). This suggests that mismatches did not result from low sequencing depth but might have arisen because of cross-barcoding. Cross-barcoding occurs during library preparation when barcode adapters are indiscriminately ligated to DNA fragments after the samples are pooled. This could result in misinterpreting data and potentially generating false results for samples on the same run. A previous study comparing library preparation methods from ONT found that Native Barcoding library preparation provided the highest total number of reads, followed closely by Rapid Barcoding, which is consistent with our findings (19). However, the same study also showed that even low levels of cross-barcoding during library preparation led to “barcode leakage” during demultiplexing, which increased misidentified single nucleotide variants compared with non-barcoded runs. The updated Kit 14 chemistry (Native Barcoding 114.96 vs. previous kit 10 Native Barcoding 110.96) used in our study eliminated thermal inactivation of the barcode ligation enzymes, which could increase the chance of cross-barcoding. Rapid Barcoding uses a heat-activated transposase, which is inactive at room temperature, so there is little risk of cross-barcoding. Thus, we suspect that cross-barcoding during Native Barcoding library preparation contributed to a low proportion of correctly identified strain types.

The shortest turnaround time was achieved with the ONT Rapid Barcoding workflow (ONT Experiment One), which was less than 20 h. This optimal workflow takes 1 h for multiplex PCR, 1.5 h for Rapid Barcoding library preparation, 16 h for sequencing runtime, and 1 h for data analysis. This is promising for epidemiological applications, such as outbreak scenarios, where timely strain identification is critical (28). The ONT Rapid workflow delivered the strain type in less than 20 h, while Illumina took more than 50 h and failed to capture the full length of the *rpoB* target. A comparison between ONT and Illumina sequencing methods for diagnostic purposes found that the shortest turnaround time of ONT sequencing was of significant clinical value and was more important to the clinical outcome than the relatively insignificant difference in accuracy between ONT and Illumina sequences (28). We designed the bioinformatics analysis pipeline to be run using a laptop (10 CPUs, 32Gb memory) and to be user-friendly for professionals without a bioinformatics background or minimally equipped laboratories.

The per-sample cost of library preparation for ONT sequencing varied by methods. Ligation-based library preparation kits, such as the Native Barcoding kit used in Experiments Two and Three, require costly third-party reagents for end-repair, dA-tailing, and adapter ligation. For Experiments Two and Three (Native Barcoding, R10.4.1 flow cell), the library preparation and flow cell cost were approximately $10.42 USD per sample for 12 or more samples during the experiment. In contrast, the Rapid Barcoding kit used in Experiment One did not require extra reagents and was approximately $6.62 USD per sample for 12 or more samples during the experiment. ONT methods are less expensive than the estimated $26.14 per sample for Illumina library preparation.

We also washed and reused minION flow cells to decrease costs and found that the read quality was not impacted. As shown in Table 3, a subset of samples from ONT Experiment One (new flow cell) was sequenced in a second run after washing the flow cell (Experiment One/washed). The sequence types obtained from the washed and reused flow cells were identical to those of the corresponding samples in the first run using a new flow cell. Compared with another approach (13), wherein a single flow cell was successfully used five times, our results also indicate that the effects of the flow cell reuse are marginal, and sequence quality is not influenced by the preceding run. Decreased pore counts following each wash should be accounted for, and we suggest adjusting runtime to reach a minimum of 50x coverage for all loci in each sample.

The Rapid ONT workflow developed in this study generated highly accurate sequences. However, some inherent errors may still exist due to error-prone ONT reads. A recent proof of concept for ONT amplicon sequencing called 97% of expected variants and noted a high error rate, especially for homopolymer and homopolymer-adjacent regions (17). We corrected similar homopolymer errors by using Medaka polishing. Alignment of IGS showing misidentified bases always resulted from T8 homopolymers called T7 at position 113. These were manually corrected since no strain types carry a T7 homopolymer region at position 113. This manual correction of homopolymers decreases automation of the method, and therefore, more hands-on time is required to check for homopolymer errors.

It was anticipated that pooling equimolar quantities for each PCR amplicon would result in comparable average depth for each product when aligned to the respective reference. However, the average depth for each amplicon varied widely between 32 and 16,776x (mean = 16,230 std. err = 24,249) across ONT Experiments One, Two, and Three. This result is comparable to another group which noted a range of 127- to 19,626-fold coverage (mean = 8320.69, std. err = 452.99) for ONT amplicon sequencing, and a minimum of 100x coverage was required for typing (17). Similarly, we found that 50x coverage of each amplicon in the multiplex was required for the optimized workflow. Setting a minimum coverage per amplicon ensures that all loci in the sample have adequate sequence information for a high-quality consensus sequence. We also found a high standard deviation of coverage between barcodes for all ONT runs (Table 3). This suggests that the sequencing run parameters can be better optimized to reduce unnecessary sequencing time by normalizing the coverage across barcodes. Barcode balancing in minKNOW normalizes coverage in real-time and could be used for future runs. The lowest per-sample coverage was observed for ONT Experiment One new flow cell with 68 samples. This outcome was consistent with the logical implications of the experimental design, in which 68 samples were sequenced for the same amount of time as subsequent runs with only 24 samples.

We recommend the use of multiplex PCR and ONT Rapid Barcoding library preparation for the typing of *M. ovipneumoniae* due to the high accuracy of the consensus sequences, lowest cost, and shortest turnaround time. These benefits are compounded when multiplexing many samples, making the workflow ideal for outbreak scenarios or population surveys (14, 29). The workflow can be implemented in-house with no initial capital, lower per-sample cost, and less technician hands-on time than Sanger or Illumina sequencing. In contrast, the initial capital cost for Illumina sequencing is often prohibitive; instead, laboratories rely on off-site commercial facilities, which may take 2 weeks for results.

A unique challenge of this study is the diversity of *M. ovipneumoniae* strain types. Only a subset of archived samples from bighorn sheep strain types was selected for this study, and we, therefore, assume that the selected samples are representative of all strain types. Furthermore, the detection of multiple strain types in one sample was not assessed in this study, although the presence of multiple strain types has previously been observed in wild and domestic sheep populations (30). Minimal modifications to our workflow would be needed to add loci or change for any MLST. Modifications of the multiplex PCR and the reference allele text file in the pipeline were the only changes required to customize this pipeline, add more loci, or other substitute MLST schemes. A limitation of the comparison of the ONT library preparation methods is the difference in technology revisions. ONT Experiment One used the R9.4.1 flow cell and ONT Experiments Two and Three used the R10.4.1 flow cells. There are a few other studies on the performance of R10.4.1/kit14 for amplicon sequencing. One group compared R9.4.1 chemistry with R10.4.0 and reported that although R10.4.0 reads were more accurate, R9.4.1 flow cells were more reliable (31). The discrepancies we noted between ONT Experiment One and ONT Experiments Two and Three could be explained by the differing flow cell and sequencing chemistry changes but not the library preparation method. Further investigation of R10.4.1 and the Rapid Barcoding kit for multiplex amplicon sequencing could eliminate the need for manual homopolymer correction, as claimed by ONT. In this study, we used a set runtime of 16 h for ONT sequencing; however, once there were 4,000 reads per sample, a similar workflow for MLST of *S. aureus* stopped sequencing, to ensure adequate coverage without oversampling (13). In that protocol, the authors used a single flow cell five times (467 samples total) and less than 4 h were required for adequate sequence data for the first three runs, with successive runs requiring 6–15 h until the flow cell was depleted (13). This approach could decrease turnaround time and cost for our workflow.

## 5 Conclusion

We developed and validated a workflow for multilocus sequence typing of *M. ovipneumoniae* directly from clinical samples using multiplex PCR and Nanopore Rapid Barcoding sequencing. This method was compared with Nanopore Native Barcoding library preparation and Illumina MiSeq-modified amplicon protocols, to determine the most accurate and cost-effective method for sequencing multiplex amplicons. Nanopore Rapid Barcoding sequencing produced the most accurate consensus sequences with the shortest workflow time. The difficulty in obtaining highly accurate consensus sequences from error-prone nanopore reads was mitigated through high coverage and consensus polishing. Therefore, the workflow is suitable for diagnostic settings, where reduced hands-on time, cost, and multiplexing capabilities are important. To the best of our knowledge, this is the first Rapid Barcoding ONT workflow developed for *Mycoplasma*, a method that could be applied to type other *Mycoplasma* species or other fastidious bacteria.

## Data availability statement

Raw sequence reads for all runs conducted in this study, and final consensus sequences are available at https://doi.org/10.6084/m9.figshare.25395310.

## Ethics statement

Ethical approval was not required for the study involving animals in accordance with the local legislation and institutional requirements because This study used retrospective archived DNA samples from nasal swabs collected from sheep. These samples were part of the routine work-up of a diagnostic laboratory. Therefore, there was no need for ethical approval.

## Author contributions

IF: Data curation, Formal analysis, Investigation, Methodology, Software, Validation, Writing – original draft. RW: Methodology, Validation, Writing – review & editing. JS: Formal analysis, Investigation, Methodology, Software, Writing – review & editing. NR: Data curation, Methodology, Visualization, Writing – review & editing. JB-M: Supervision, Visualization, Writing – review & editing. GC: Methodology, Validation, Writing – review & editing. PK: Methodology, Validation, Writing – review & editing. GM: Conceptualization, Funding acquisition, Project administration, Resources, Supervision, Visualization, Writing – review & editing.

## Footnotes

^1^ https://github.com/nanoporetech/dorado

^2^ https://jgi.doe.gov/data-and-tools/software-tools/bbtools/
