# Supplementary material for: High-throughput rapid amplicon sequencing for multilocus sequence typing of Mycoplasma ovipneumoniae from archived clinical DNA samples
Source: Front Vet Sci. 2024 Jul 31;11:1443855. doi: 10.3389/fvets.2024.1443855 (PMC11322507; doi:10.3389/fvets.2024.1443855)

**Supplementary 6 Geneious amplicon analysis instructions**

Instructions for processing consensus sequences form Illumina or Nanopore workflows

1. Setup working environment.
   1. Create a new folder using the “add” button in the toolbar.
2. Import consensus sequences by dragging and dropping them from the output directory of the pipeline.


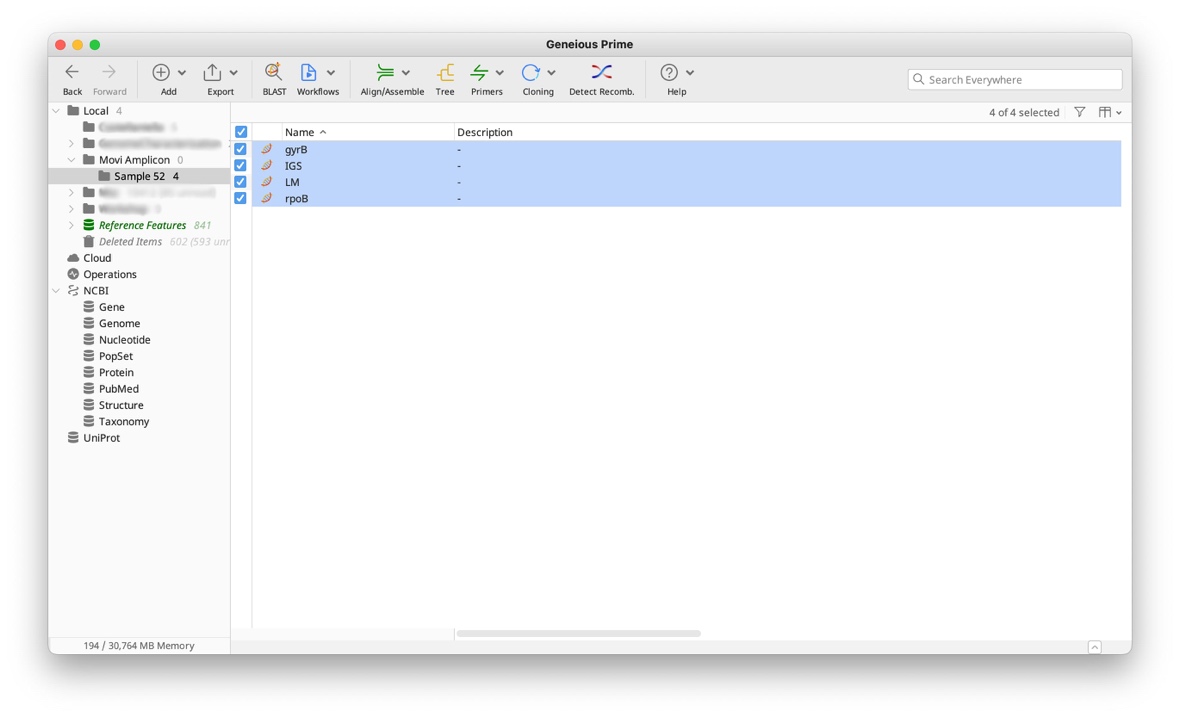


1. Concatenate consensus sequences in the following order: LM, IGS, rpoB, gyrB
   1. Select the 4 sequences.
   2. Search for “concatenate” using the search function in the toolbar and select “Concatenate Sequences or Alignments”.
   3. Adjust the order of sequences


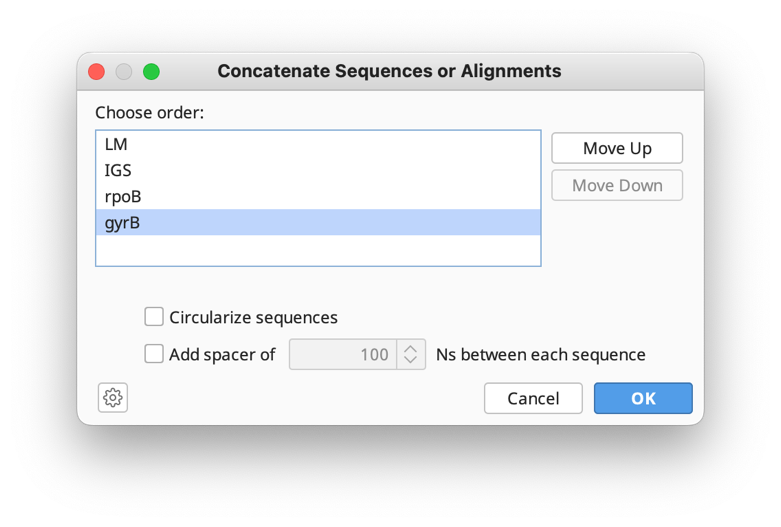

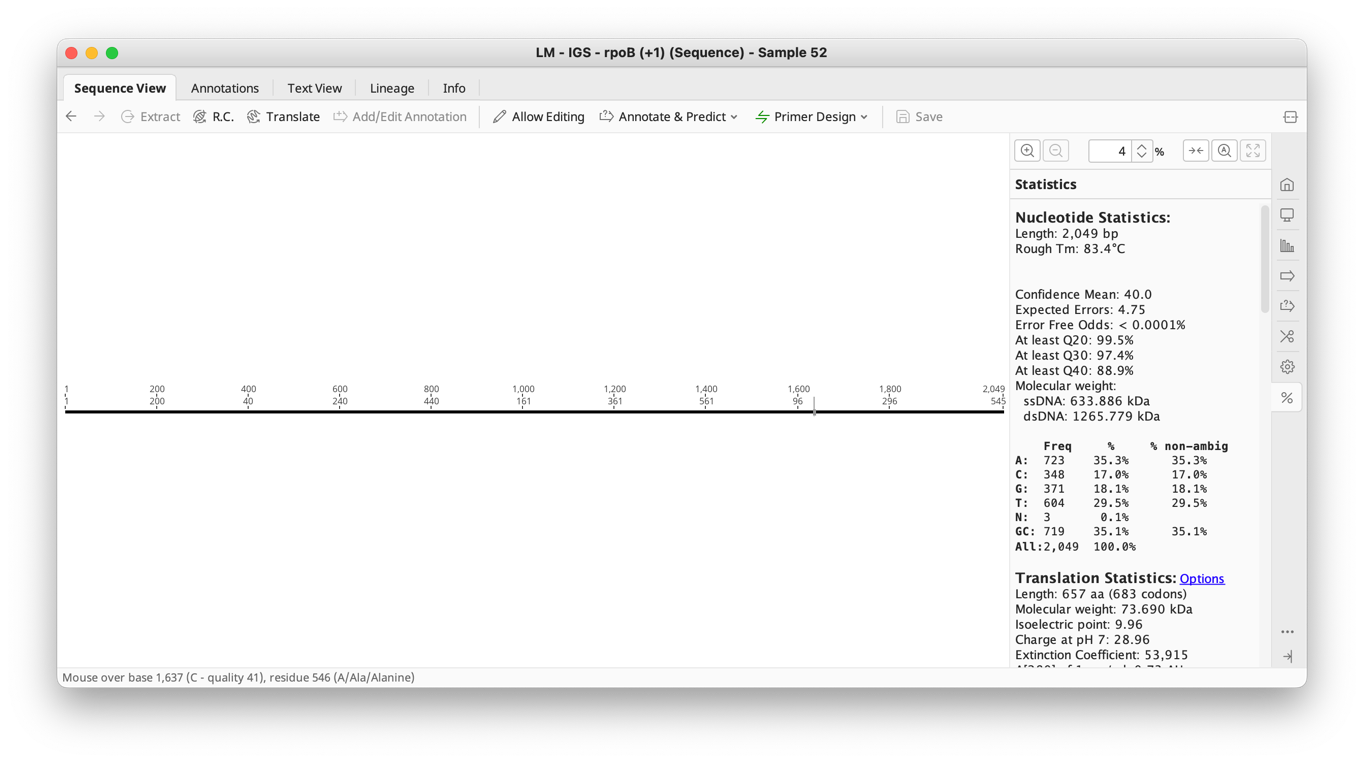


1. Create blast database (for first time use of the pipeline).
   1. Download all reference sequences in Geneious via NCBI accession numbers listed in Supplementary 10. Concatenate sequences as required for each strain types in the table. Select all the concatenated reference the sequences.
   2. From the menu bar, select Tools > BLAST Add/Remove dredatabases > Add BLAST Database.


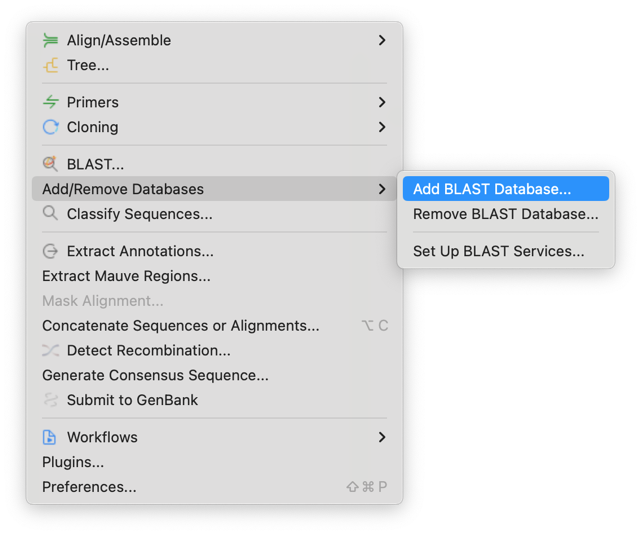

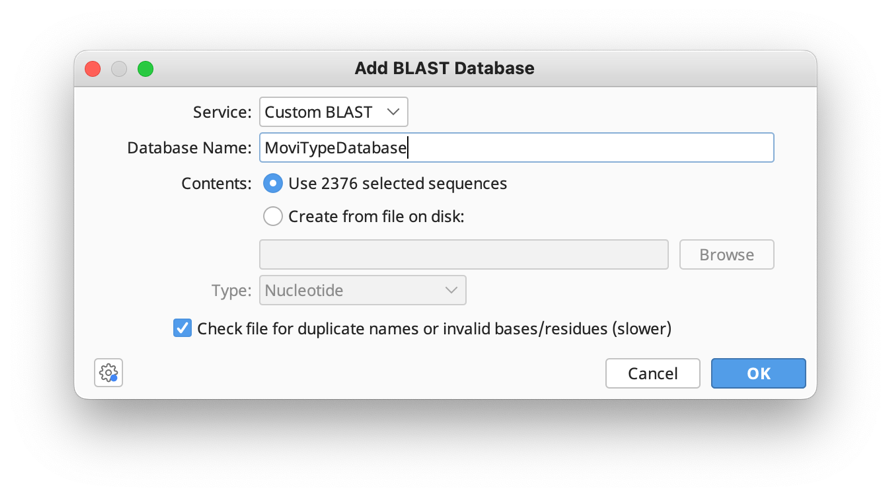


1. Search the database for similar sequences/types
   1. Select the concatenated sequence for the desired samnple.
   2. Click BLAST icon in the toolbar and select the custom database created in step 4
   3. Run BLAST
   4. Manually check the top result for alignment agreement.


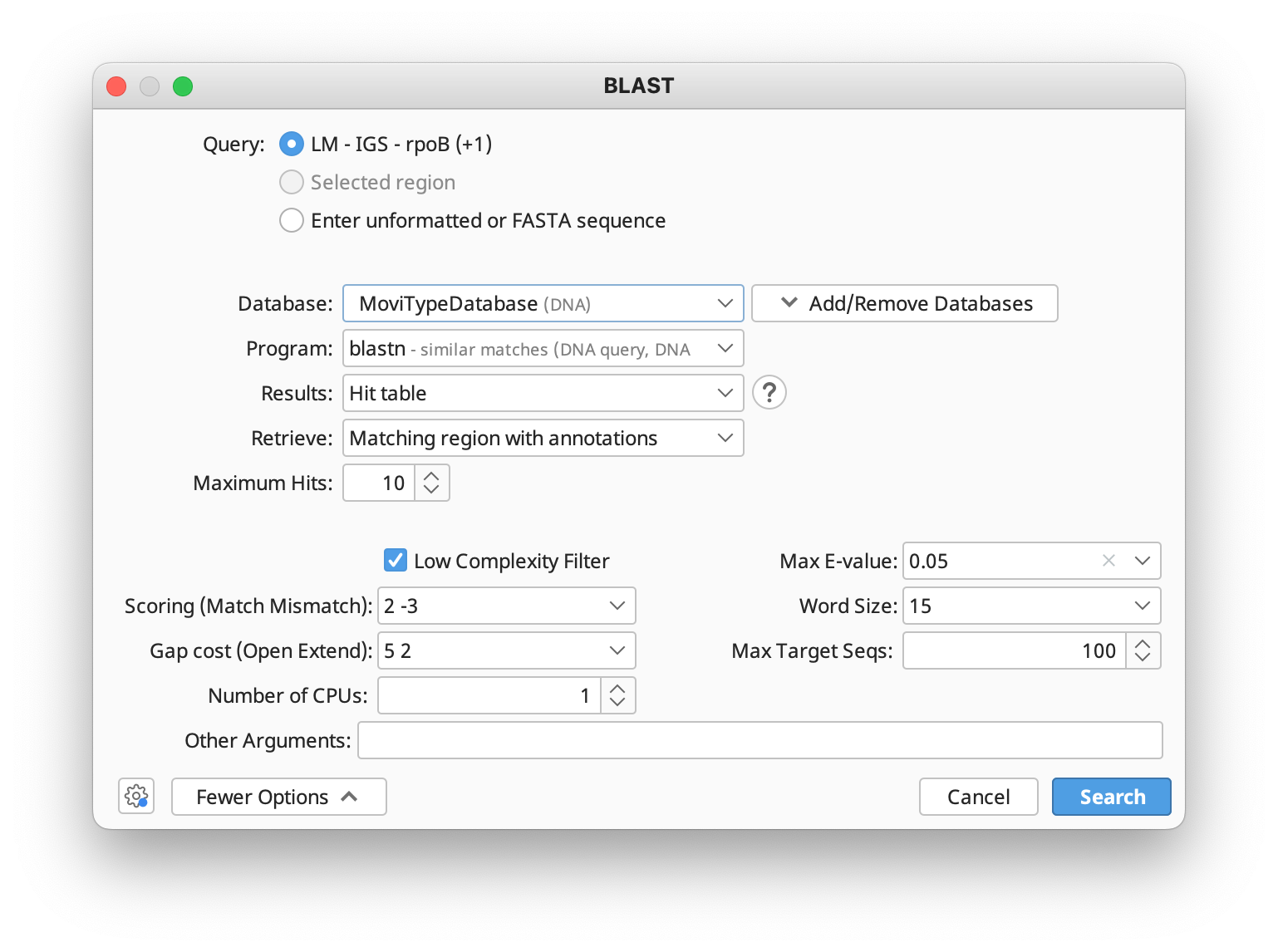

Supplement: Supplementary file 1 [file Data_Sheet_1.ZIP › Supplementary_corrected/Supplementary 6 Geneious amplicon analysis insructions.docx]
