# Supplementary material for: High-throughput rapid amplicon sequencing for multilocus sequence typing of Mycoplasma ovipneumoniae from archived clinical DNA samples
Source: Front Vet Sci. 2024 Jul 31;11:1443855. doi: 10.3389/fvets.2024.1443855 (PMC11322507; doi:10.3389/fvets.2024.1443855)
Supplement: Supplementary file 1 [file Data_Sheet_1.ZIP › Supplementary_corrected/Supplementary 3 Multiplex PCR.docx]

**Supplementary 3. Multiplex PCR**

**Supplementary 3A.** Multiplex PCR cycle conditions for 35 cycles with a 50µl reaction size.

| **Step** | **Temperature (℃)** | **Time (s)** |
| --- | --- | --- |
| Initial | 98 | 10 |
| Denature | 98 | 3 |
| Anneal | 49 | 10 |
| Extend | 72 | 25 |
| Final | 72 | 120 |
| Hold | 4 | - |

**Supplementary 3B.** Multiplex PCR reaction components by volume.

| **Component** | **Volume (µL)** |
| --- | --- |
| Phusion 2x | 25.0 |
| H_2_O | 2.0 |
| LM | 4.0 |
| IGS | 1.5 |
| *rpoB* | 1.0 |
| *gyrB* | 2.0 |
| Ex-IGS | 0.5 |
| Ex-*rpoB* | 0.5 |
| Ex-*gyrB* | 1.0 |
| Sample DNA | 2.0 |
| Total: | 50.0 |

Primer volumes assume a 10 mM stock solution. Ex- denotes external primer pair. LM=16S rDNA gene, IGS=16-23S intergenic spacer region, *rpoB*=RNA polymerase beta subunit gene, *gyrB*=gyrase beta subunit gene. Phusion 2x=Phusion Flash High-Fidelity PCR Master Mix (ThermoFisher cat. F548)

**Supplementary 3C**. Gel electrophoresis of the optimized multiplex PCR assay showing amplification of four gene fragments from *Mycoplasma ovipneumoniae* from DNA samples (throat washes, lung tissue swab, or nasal swab). DNA marker 100-1000 bp in 100 bp increments. IGS: 16-23S intergenic spacer region; rpoB: RNA polymerase beta subunit gene; gyrB: DNA gyrase beta subunit gene; LM= 16s rDNA. Gel electrophoresis conducted using 2.0% agarose in a 1X lithium acetate borate buffer solution. Gel was run for 1.5 hours at 120V. Gel Imaged using a GelDoc Go system (BioRad), using faint bands protocol.


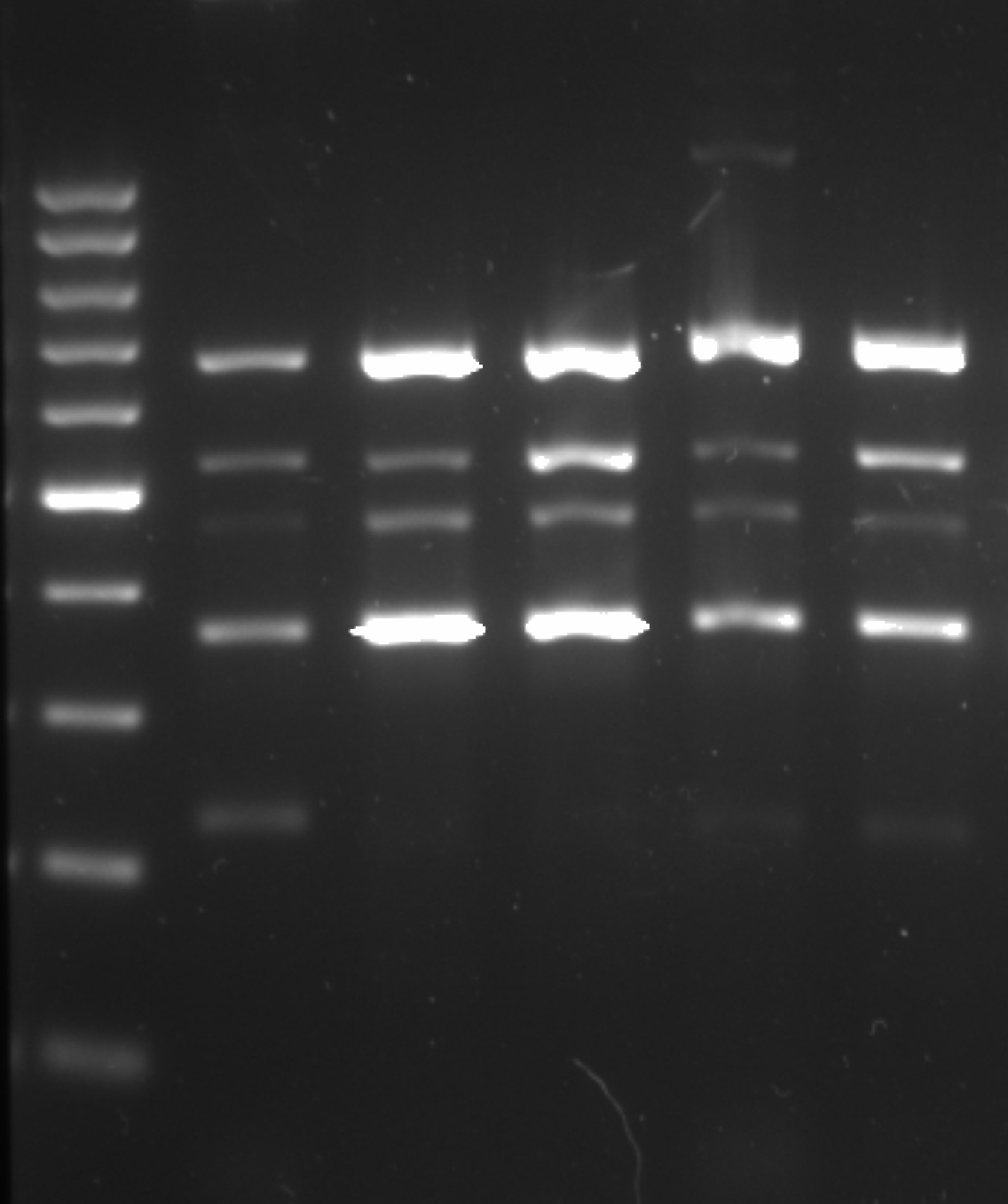


**500 bp**

**100 bp**

**1000 bp**

***rpoB –* 680 bp**

***gyrB –* 547 bp**

**IGS – 470-510 bp**

**LM – 361 bp**

**DNA marker**

**50**

**52**

**54**

**55**

**57**
