# Supplementary material for: High-throughput rapid amplicon sequencing for multilocus sequence typing of Mycoplasma ovipneumoniae from archived clinical DNA samples
Source: Front Vet Sci. 2024 Jul 31;11:1443855. doi: 10.3389/fvets.2024.1443855 (PMC11322507; doi:10.3389/fvets.2024.1443855)
Supplement: Supplementary file 1 [file Data_Sheet_1.ZIP › Supplementary_corrected/Supplementary file 7 depth.docx]

Supplementary file 7 – Average sequence depth of loci from ONT sequencing runs.

**Supplementary 7A:** Average sequence depth of loci in ONT and Illumina sequencing runs post-filtering with all samples. Depth is reported as the number of times a base is read averaged across each locus, ± the standard deviation for the sequencing run.

| **Experiment no.** | ***gyrB*** | **IGS** | **LM** | ***rpoB*** |
| --- | --- | --- | --- | --- |
| Experiment 1 new | 3315 ± 2274 | 2617 ± 1908 | 1493 ± 1158 | 264 ± 257 |
| Experiment 1 washed | 3164 ± 2329 | 7903 ± 9890 | 5185 ± 4164 | 504 ± 407 |
| Experiment 2 new | 104 ± 38 | 10985 ± 10241 | 6782 ± 6459 | 28844 ± 14112 |
| Experiment 3 new | 271 ± 66 | 39208 ± 35628 | 27336 ± 20740 | 63146 ± 46643 |
| Experiment 3 washed | 80 ± 31 | 16276 ± 15525 | 14774 ± 12174 | 21537 ± 16771 |

**Supplementary 7B:** Average sequence depth of loci from ONT sequencing runs
