# Supplementary material for: High-throughput rapid amplicon sequencing for multilocus sequence typing of Mycoplasma ovipneumoniae from archived clinical DNA samples
Source: Front Vet Sci. 2024 Jul 31;11:1443855. doi: 10.3389/fvets.2024.1443855 (PMC11322507; doi:10.3389/fvets.2024.1443855)
Supplement: Supplementary file 1 [file Data_Sheet_1.ZIP › Supplementary_corrected/Supplementary 2 Singleplex PCR.docx]

**Supplementary 2 Singleplex PCR**

**Supplementary 2A.** Primer sequences for *Mycoplasma ovipneumoniae* multi locus sequence typing.

| **Target** | **Direction** | **Nesting** | **Primer sequence 5’ to 3’** |
| --- | --- | --- | --- |
| LM | Forward | - | TGAACGGAATATGTTAGCTT |
| LM | Reverse | - | GACTTCATCCTGCACTCTGT |
| Ex-IGS | Forward | External | GTTAACCTCGGAGACCATTG |
| Ex-IGS | Reverse | External | GTTTGCTAGGTTGGGTTTCC |
| IGS | Forward | Internal | GGAACACCTCCTTTCTACGG |
| IGS | Reverse | Internal | CCAAGGCATCCACCAAATAC |
| Ex-*rpoB* | Forward | External | AGTTATCACAATTTATGGATCAAA |
| Ex-*rpoB* | Reverse | External | GCTCAAAGTTCCATTTCNCCGAA |
| *rpoB* | Forward | Internal | TCGGCTTCAGCAATTCCTTTCTT |
| *rpoB* | Reverse | Internal | TCGGCTGTTGGGTTGTCTTCTC |
| Ex-*gyrB* | Forward | External | AAAACGWCCAGGKATGTATATTGG |
| Ex-*gyrB* | Reverse | External | GGATCCATTGTTGTTTCTCATAATTG |
| *gyrB* | Forward | Internal | GGGTCAAACAAAAGCAAAACTAAA |
| *gyrB* | Reverse | Internal | ACGGAATAAAAATGTCAAAAGTAA |

LM reaction not nested. ‘Ex-’ = external. Primer sequences developed as part of a previous study: Cassirer, E. F., Manlove, K. R., Plowright, R. K., & Besser, T. E. (2017). Evidence for strain-specific immunity to pneumonia in bighorn sheep. *The Journal of Wildlife Management*, *81*(1), 133-143. https://doi.org/10.1002/jwmg.21172.

**Supplementary 2B.** Singleplex and nested singleplex PCR cycle conditions and reaction volumes. “Ex-” denotes external primer pair. IGS=16-23S intergenic spacer region, *rpoB*=RNA polymerase beta subunit, *gyrB*=DNA gyrase beta subunit, LM=16s rDNA. Primer volumes assume a 10 mM stock solution.

| Step |  | Ex-IGS | |  | Ex-*rpoB* | |  | Ex-*gyrB* | |  | LM | |
| --- | --- | --- | --- | --- | --- | --- | --- | --- | --- | --- | --- | --- |
|  |  | Temp. (°C) | Time (s) |  | Temperature (°C) | Time (s) |  | Temp. (°C) | Time (s) |  | Temp. (°C) | Time (s) |
| **Initial** |  | 98 | 10 |  | 98 | 10 |  | 98 | 10 |  | 98 | 10 |
| **Denaturation** |  | 98 | 3 |  | 98 | 3 |  | 98 | 3 |  | 98 | 3 |
| **Annealing** |  | 54 | 10 |  | 50 | 10 |  | 50 | 10 |  | 48 | 10 |
| **Extension** |  | 72 | 20 |  | 72 | 30 |  | 72 | 30 |  | 72 | 10 |
| **Final** |  | 72 | 120 |  | 72 | 120 |  | 72 | 120 |  | 72 | 120 |
|  |  |  |  |  |  |  |  |  |  |  |  |  |
| Step |  | IGS | |  | *rpoB* | |  | *gyrB* | |  |  |  |
|  |  | Temp. (°C) | Time (s) |  | Temp. (°C) | Time (s) |  | Temp. (°C) | Time (s) |  |  |  |
| **Initial** |  | 98 | 10 |  | 98 | 10 |  | 98 | 10 |  |  |  |
| **Denaturation** |  | 98 | 3 |  | 98 | 3 |  | 98 | 3 |  |  |  |
| **Annealing** |  | 52 | 10 |  | 50 | 10 |  | 50 | 10 |  |  |  |
| **Extension** |  | 72 | 15 |  | 72 | 15 |  | 72 | 15 |  |  |  |
| **Final** |  | 72 | 120 |  | 72 | 120 |  | 72 | 120 |  |  |  |

**Supplementary 2C**. PCR reaction components by volume for *Mycoplasma ovipneumoniae* multi locus sequence typing.

| **Component** | **Volume (µL)** |
| --- | --- |
| Phusion 2x | 10.0 |
| H_2_O | 7.0 |
| Forward primer | 1.0 |
| Reverse primer | 1.0 |
| Sample DNA | 1.0 |
| Total: | 20.0 |

Primer volumes assume a 10 mM stock solution.

Phusion 2x=Phusion Flash High-Fidelity PCR Master Mix (ThermoFisher cat. F548)

**Supplementary 2D**. Singleplex PCR gel image. A 2% agarose gel was prepared in-house using a 1X lithium acetate borate buffer solution (Sigma-Aldrich, Burlington, MA, USA), SYBR safe DNA gel stain (Invitrogen, Waltham, MA, USA) and a 100 to 1000 bp DNA marker (Invitrogen, Waltham, MA, USA). All samples were loaded using TriTrack loading dye (ThermoFisher Scientific, Waltham, MA, USA). Gels were run in the buffer solution at 120 V, then imaged using a GelDoc Go (Bio-Rad, Hercules CA, USA). IGS=16-23S intergenic spacer region, *rpoB*=RNA polymerase beta subunit, *gyrB*=DNA gyrase beta subunit, LM=16s rDNA. ‘Ex-’ = external.


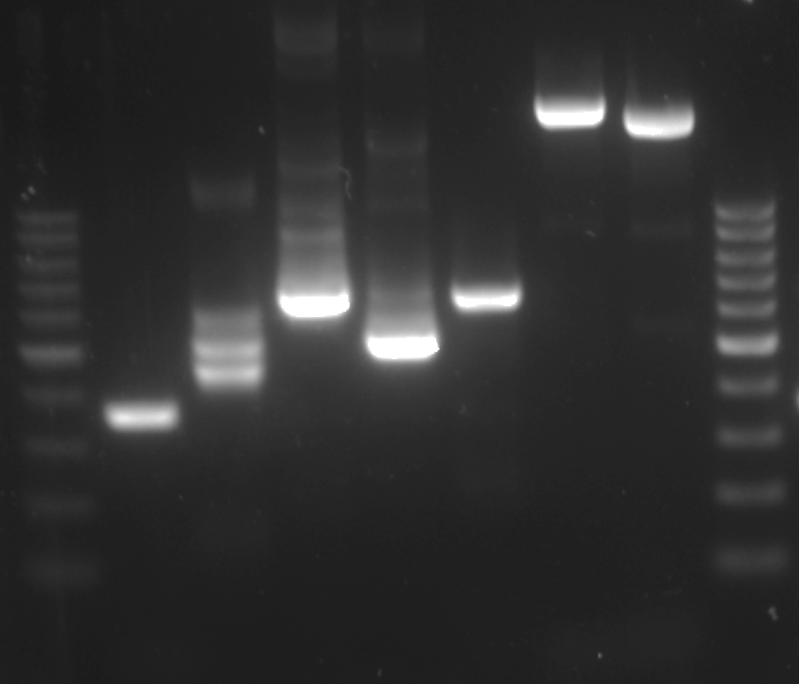


LM

IGS

*rpoB*

*gyrB*

Ex-IGS

Ex-*rpoB*

Ex-*gyrB*

500 bp

DNA marker

DNA marker

500 bp

100 bp

100 bp

1000 bp

1000 bp
