# Supplementary material for: High-throughput rapid amplicon sequencing for multilocus sequence typing of Mycoplasma ovipneumoniae from archived clinical DNA samples
Source: Front Vet Sci. 2024 Jul 31;11:1443855. doi: 10.3389/fvets.2024.1443855 (PMC11322507; doi:10.3389/fvets.2024.1443855)
Supplement: Supplementary file 1 [file Data_Sheet_1.ZIP › Supplementary_corrected/Supplementary 5 Illumina primers.docx]

**Supplementary 5 Illumina primers**

**Supplementary 5.** Illumina sequencing adapter overhang seqeunces and *M. ovipneumoniae* MLST internal primers.

| **Name** | **Locus-specific primer** | **Overhang adapter with locus specific primer 5’ to 3’** |
| --- | --- | --- |
| LM-F | TGAACGGAATATGTTAGCTT | TCGTCGGCAGCGTCAGATGTGTATAAGAGACAGTGAACGGAATATGTTAGCTT |
| LM-R | GACTTCATCCTGCACTCTGT | GTCTCGTGGGCTCGGAGATGTGTATAAGAGACAGGACTTCATCCTGCACTCTGT |
| IGS-F | GGAACACCTCCTTTCTACGG | TCGTCGGCAGCGTCAGATGTGTATAAGAGACAGGGAACACCTCCTTTCTACGG |
| IGS-R | CCAAGGCATCCACCAAATAC | GTCTCGTGGGCTCGGAGATGTGTATAAGAGACAGCCAAGGCATCCACCAAATAC |
| rpoB-F | TCGGCTTCAGCAATTCCTTTCTT | TCGTCGGCAGCGTCAGATGTGTATAAGAGACAGTCGGCTTCAGCAATTCCTTTCTT |
| rpoB-R | TCGGCTGTTGGGTTGTCTTCTC | GTCTCGTGGGCTCGGAGATGTGTATAAGAGACAGTCGGCTGTTGGGTTGTCTTCTC |
| gyrB-F | GGGTCAAACAAAAGCAAAACTAAA | TCGTCGGCAGCGTCAGATGTGTATAAGAGACAGGGGTCAAACAAAAGCAAAACTAAA |
| gyrB-F | ACGGAATAAAAATGTCAAAAGTAA | GTCTCGTGGGCTCGGAGATGTGTATAAGAGACAGACGGAATAAAAATGTCAAAAGTAA |

Custom amplicon primers were prepared for use with the 16S Metagenomic Library Preparation illumina for Illumina MiSeq System library preparation protocol (Illumina Part # 15044223 Rev. A, <https://support.illumina.com/documents/documentation/chemistry_documentation/16s/16s-metagenomic-library-prep-guide-15044223-b.pdf>). Locus-specific primer sequences were appended with adapter overhang sequences as detailed in the “Amplicon primers” section on page three of the Illumina protocol document. Forward: TCGTCGGCAGCGTCAGATGTGTATAAGAGACAG; Reverse: GTCTCGTGGGCTCGGAGATGTGTATAAGAGACAG

LM=16S rDNA gene, IGS=16-23S intergenic spacer region, *rpoB*=RNA polymerase beta subunit gene, *gyrB*=gyrase beta subunit gene. Locus-specific primer sequences developed as part of a previous study: Cassirer, E. F., Manlove, K. R., Plowright, R. K., & Besser, T. E. (2017). Evidence for strain-specific immunity to pneumonia in bighorn sheep. *The Journal of Wildlife Management*, *81*(1), 133-143. <https://doi.org/10.1002/jwmg.21172>.
